# Supplementary material for: Elucidation of 4-Hydroxybenzoic Acid Catabolic Pathways in Pseudarthrobacter phenanthrenivorans Sphe3
Source: Int J Mol Sci. 2024 Jan 10;25(2):843. doi: 10.3390/ijms25020843 (PMC10815724; doi:10.3390/ijms25020843)
Supplement: Supplementary file 1 [file ijms-25-00843-s001.zip › ijms-2795990-supplementary.pdf]

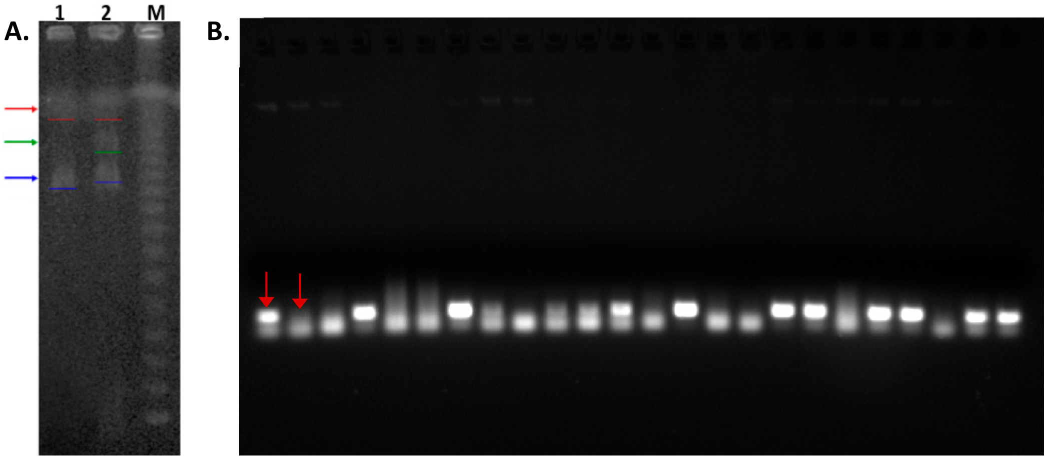

Figure S1: A. Pulsed field electrophoresis agarose gel from Sphe and Sphe3c cells. 1: DNA from the mutant Sphe3c cells; 2: DNA from the wild-type Sphe3 cells; M: λDNA ladder (Chef DNA Size Standard, 48.5-1000 kb, Bio-Rad). Red arrow and lines designate the chromosomal DNA band, green arrow and line designate the pASPHE301 plasmid bands and blue designates the pASPHE302 plasmid bands. It is obvious that the band corresponding to pASPHE301 plasmid is not present in the DNA extracted from the mutant strain, Sphe3c. **B. Agarose gel electrophoresis for PCR products with primers specific for the gene of catechol 2,3-dioxygenase**. The gene for catechol 2,3-dioxygenase is unique for the pASPHE301 plasmid and the absence of a PCR product indicates the loss of the plasmid. The primers used are the primers used for the RT-PCR experiments. The template DNA was extracted from Sphe3 cells at the end of plasmid-curing procedure.

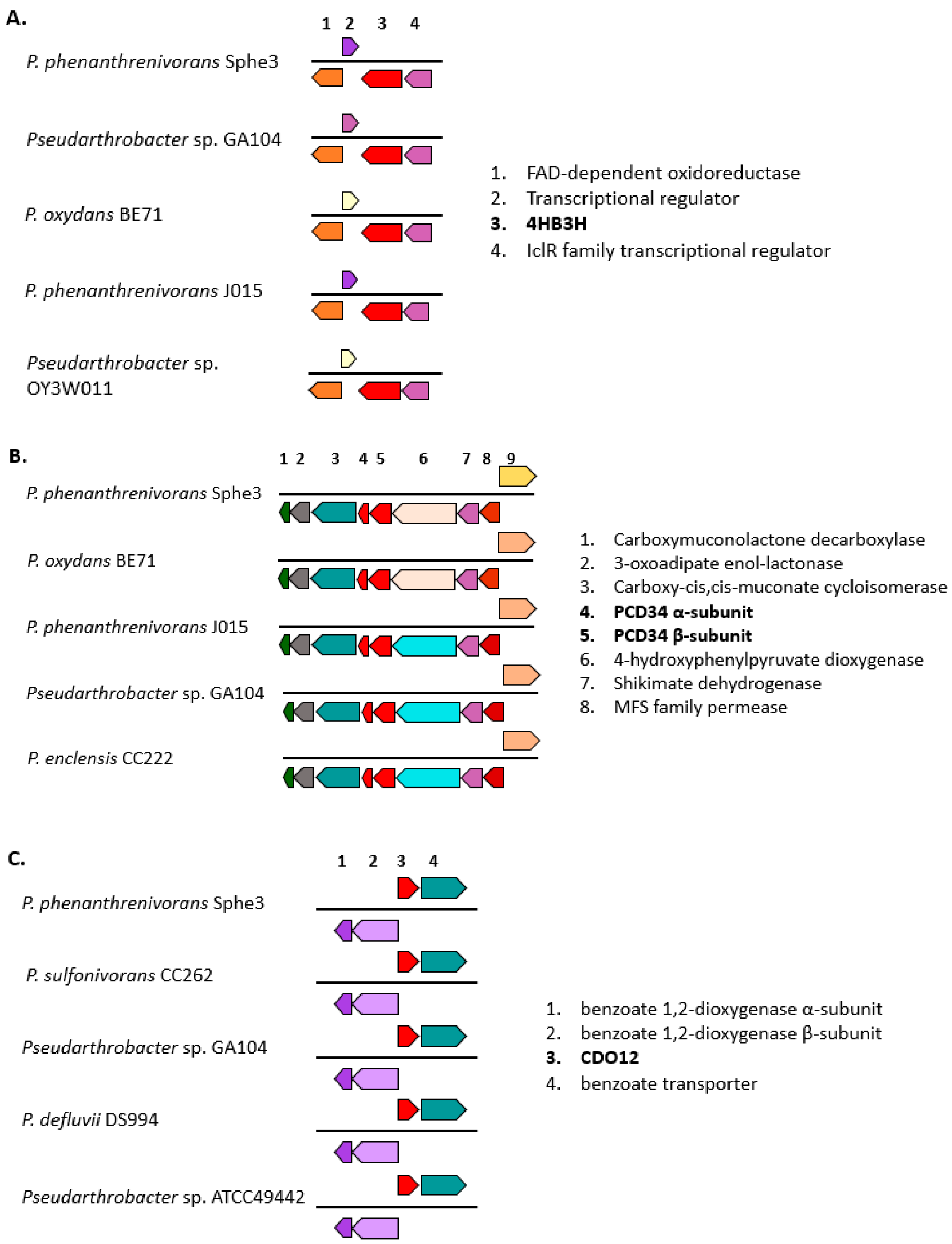

Figure S2: Graphical representation of catabolic gene clusters of Sphe3 involved in 4-HBA catabolism, in alignment with the respective areas in other *Pseudarthrobacter* species. The gene of interest is colored in red. There are no data for PCA 4,5-dioxygenase (PCD45) and catechol 2,3-dioxygenase (CDO23) for other *Pseudarthrobacter* according to JGI database. The clusters depicted are of **A.** 4-hydroxybenzoate-3-hydroxylase (4HB3H), **B.** PCA 3,4-dioxygenase (PCD34) and **C.** catechol 1,2-dioxygenase (CDO12). The data were extracted from the Joint Genomic Institute (JGI) database (https://img.jgi.doe.gov/cgi-bin/m/main.cgi?section=GeneSearch&page=searchForm).

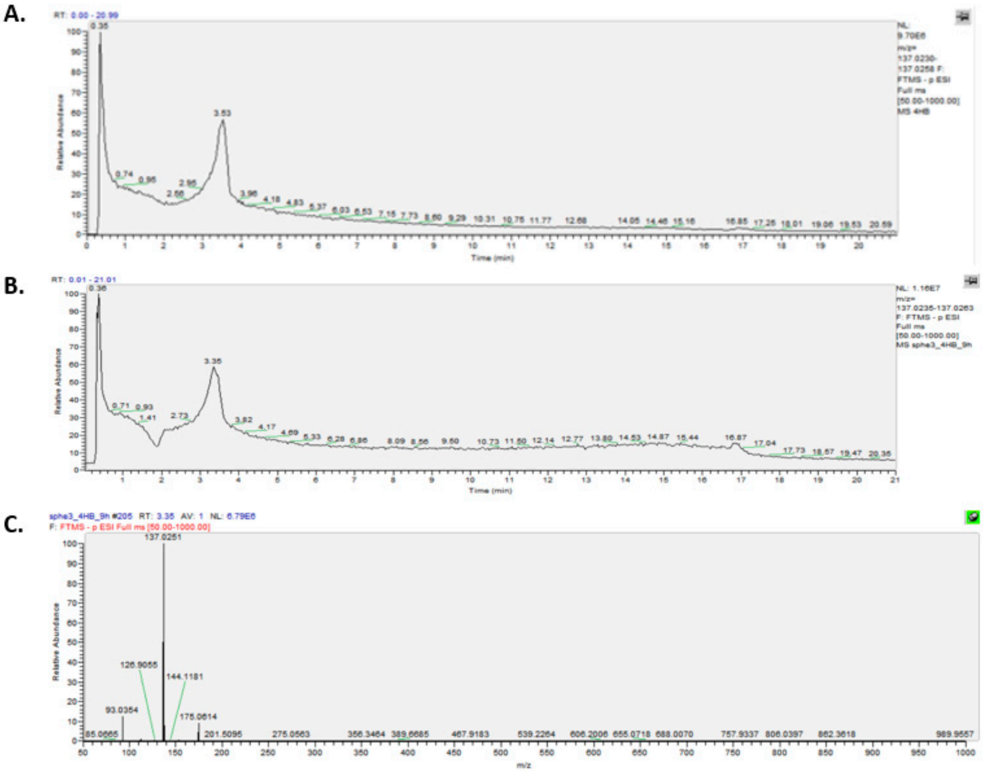

Figure S3: A. Chromatogram of the 4-HBA standard (R.T. = 3.53 min); **B.** Chromatogram of the compound identified as 4-HBA (R.T. = 3.35 min) in Sphe3 and Sphe3c cultures in 4-HBA; **C.** MS spectrum of m/z = 137.0244.

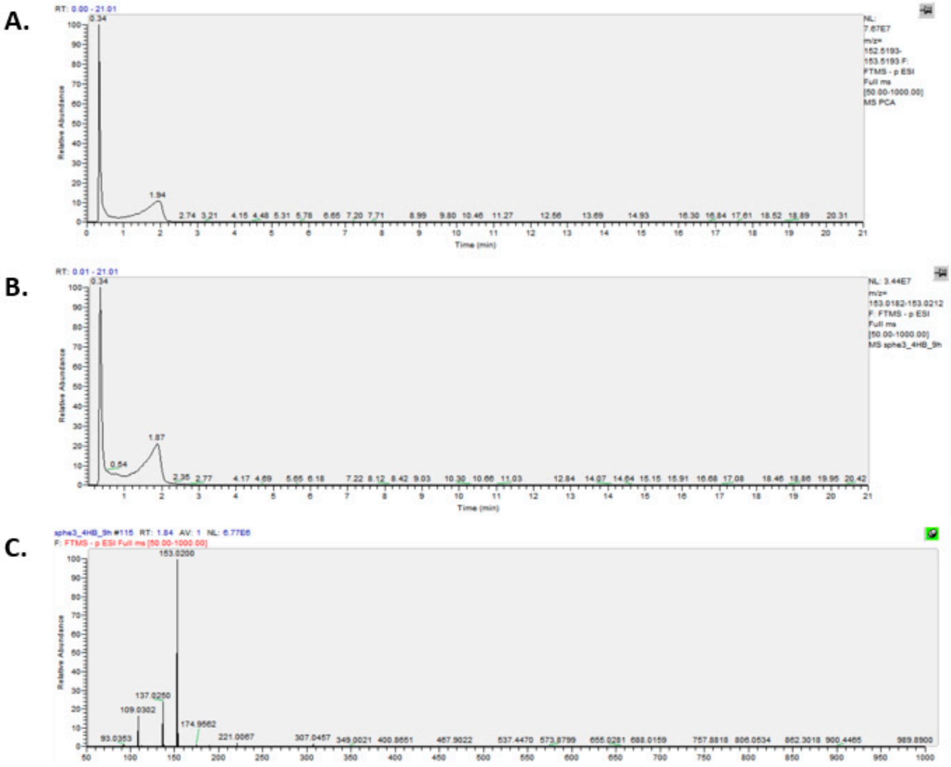

Figure S4: A. Chromatogram of the PCA standard (R.T. = 1.94 min); **B.** Chromatogram of the compound identified as PCA (R.T. = 1.88 min) in Sphe3 and Sphe3c cultures in 4-HBA; **C.** MS spectrum of m/z = 153.0193.

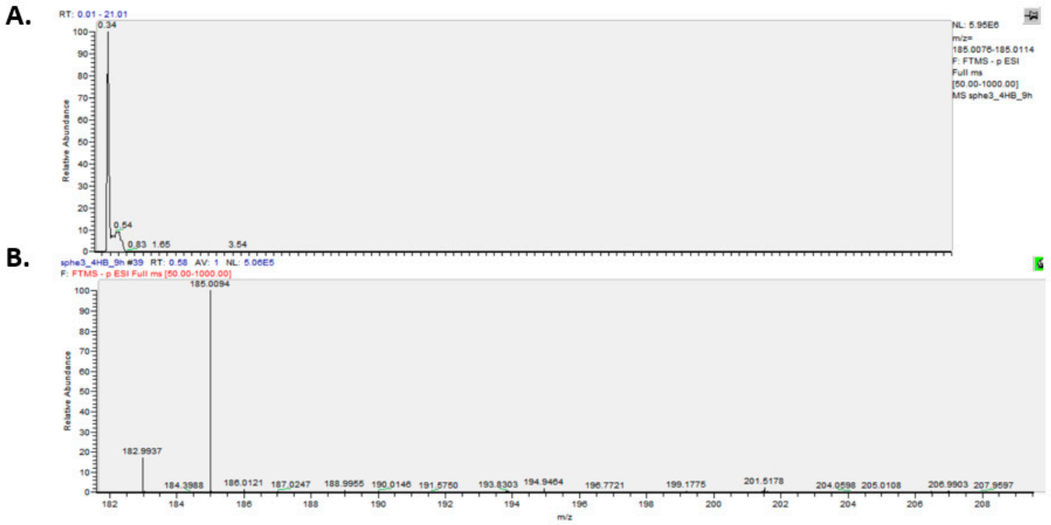

Figure S5: A. Chromatogram of the compound with m/z = 185.0091 (R.T. = 0.54 min) in Sphe3 and Sphe3c cultures in 4-HBA; **B.** MS spectrum of the compound.

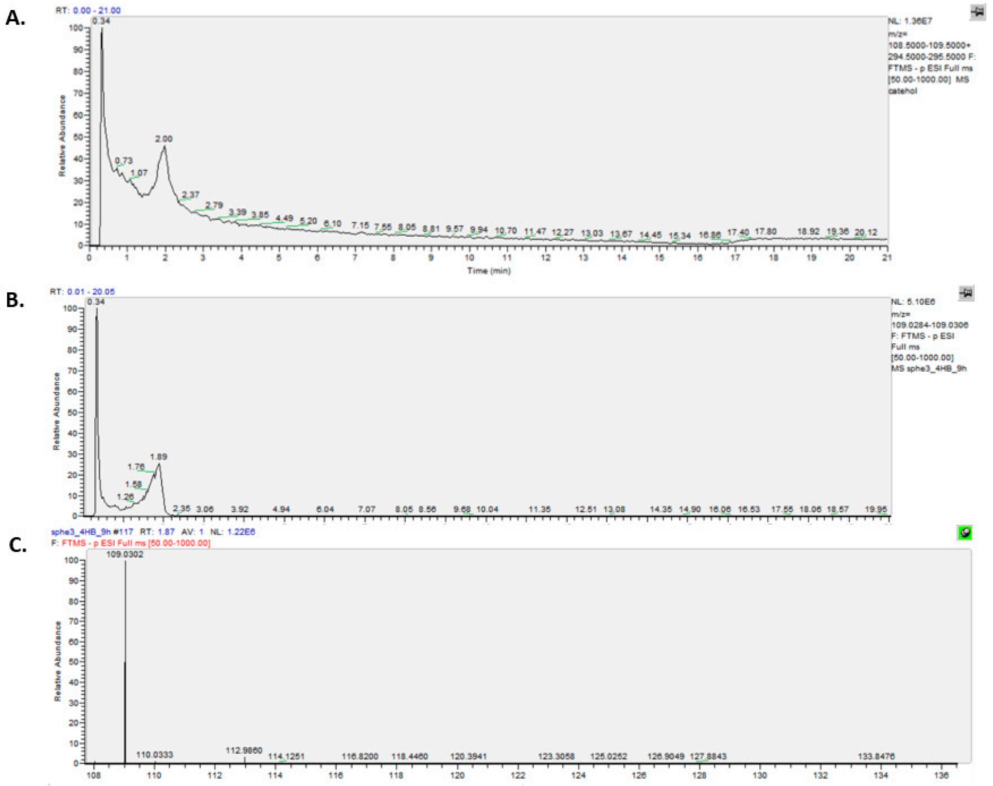

Figure S6: A. Chromatogram of the catechol standard (R.T. = 2 min); **B.** Chromatogram of the compound identified as catechol (R.T. = 1.89 min) in Sphe3 and Sphe3c cultures in 4-HBA; **C.** MS spectrum of m/z = 109.0295.

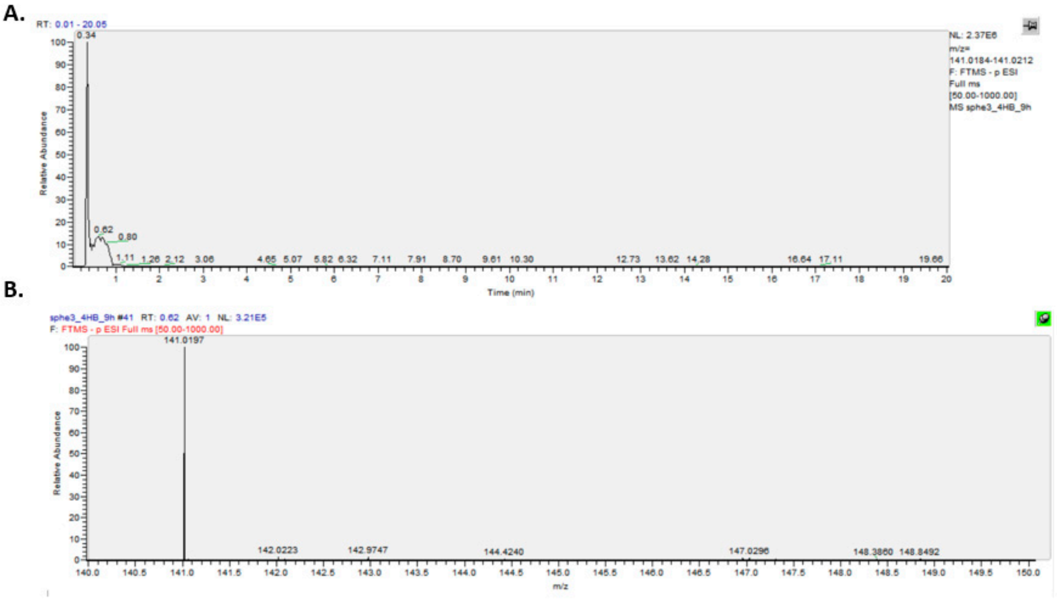

Figure S7: A. Chromatogram of the compound with m/z = 141.0193 (R.T. = 1.34 min) in Sphe3 and Sphe3c cultures in 4-HBA; **B.** MS spectrum of the compound.

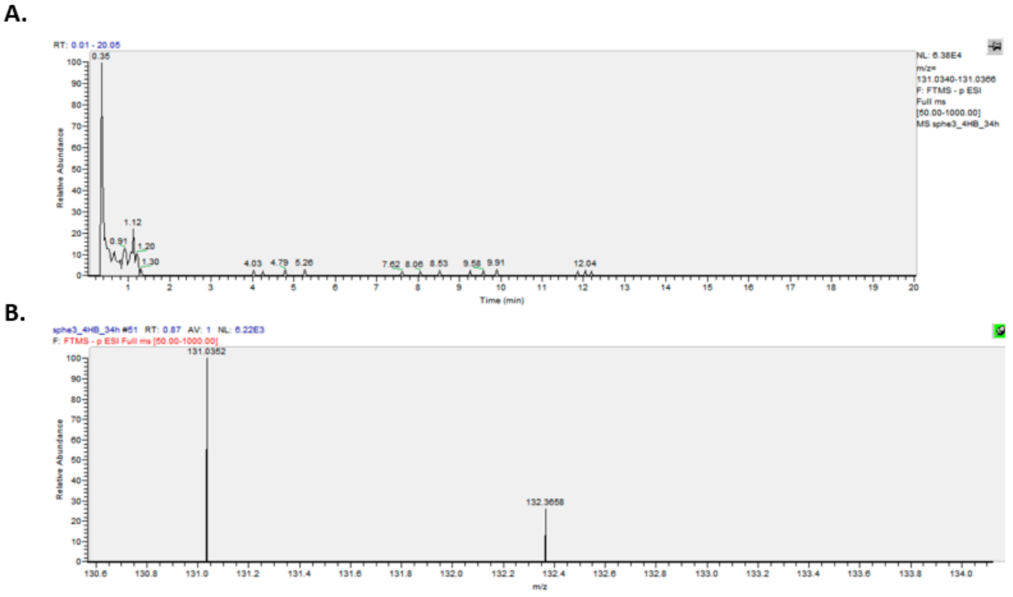

Figure S8: A. Chromatogram of the compound with m/z = 131.0357 (R.T. = 1.2 min) in Sphe3 and Sphe3c cultures in 4-HBA, identified as 4-hydroxy-2-oxopentanoate based on the m/z value; **B.** MS spectrum of the compound.

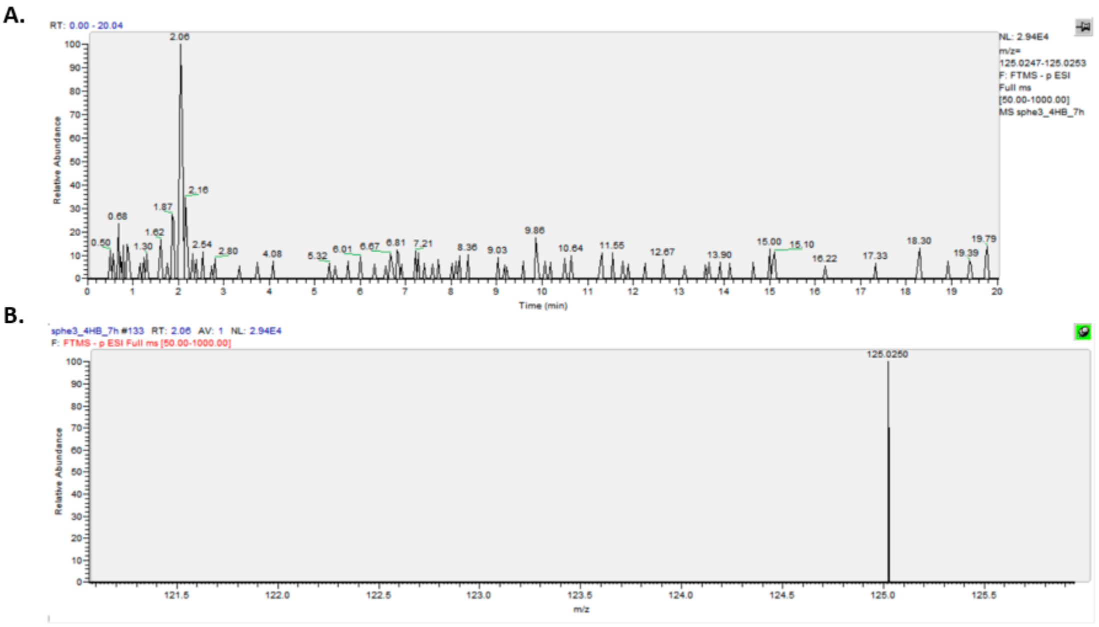

Figure S9: A. Chromatogram of the compound with m/z = 125.025 (R.T. = 2 min) in Sphe3 and Sphe3c cultures in 4-HBA, identified as hydroxyquinol based on the m/z value; **B.** MS spectrum of the compound.

Table S1: BLASTP search of enzymes that could be involved in 4-HBA degradation by *P. phenanthrenivorans* Sphe3.

Locus Tag	Description	Location	Microorganism	Query Coverage (%)	Per. Identity (%)	Length (aa)	Accession No.
Asphe3_38690	4-hydroxybenzoate-3-monooxygenase	chromosome	*Pseudarthrobacter* sp. L1SW	100	95.44	395	WP_228404732.1
			Pseudarthrobacter oxydans	100	95.18	395	WP_310113027.1
			Pseudarthrobacter scleromae	99	95.43	395	WP_188728301.1
			Pseudarthrobacter polychromogenes	99	94.92	395	WP_188810805.1
			Pseudarthrobacter siccitolerans	99	94.9	396	WP_050057210.1
			Pseudarthrobacter equi	99	93.4	395	WP_091716793.1
Asphe3_38850	PCA 3,4-dioxygenase, alpha subunit	chromosome	*Pseudarthrobacter oxydans*	100	94.12	187	WP_174177761.1
			Pseudarthrobacter scleromae	100	93.58	187	WP_188728336.1
			Pseudarthrobacter equi	100	92.51	187	WP_091716828.1
			Arthrobacter ulcerisalmonis	97	93.99	185	WP_306629419.1
			Pseudarthrobacter enclensis	97	93.44	185	WP_141944929.1
			Arthrobacter oryzae	97	92.9	185	WP_306968265.1

Asphe3_38860	PCA 3,4-dioxygenase, beta subunit	chromosome	*Pseudarthrobacter scleromae*	100	95.17	290	WP_188728339.1
			Pseudarthrobacter polychromogenes	100	94.83	290	WP_188810766.1
			Pseudarthrobacter chlorophenolicus	100	93.1	292	WP_015939000.1
			Pseudarthrobacter siccitolerans	99	93.06	295	WP_050057194.1
			Pseudarthrobacter enclensis	100	92.07	291	WP_058269269.1
			Pseudarthrobacter sulfonivorans	100	91.41	291	WP_309819042.1
Asphe3_42380	PCA 4,5-dioxygenase	pASPHE302	*Arthrobacter* sp. FB24	100	99.54	433	WP_011689793.1
			Arthrobacter sp. YJM1	99	94.65	439	WP_305997201.1
			Pseudarthrobacter sulfonivorans	99	90.93	465	WP_255769043.1
			Microbacterium resistens	99	90.49	443	WP_310020541.1
			Microbacterium hydrocarbonoxydans	99	90.93	450	WP_045257717.1
			Corynebacterium cyclohexanicum	99	89.77	465	WP_229231144.1
Asphe3_35170	catechol 1,2-dioxygenase	chromosome	*Arthrobacter* sp. BB-1	100	92.62	298	TNB68529.1
			Pseudarthrobacter sp. GA104	100	91.95	298	MUU73784.1

			Pseudarthrobacter sulfonivorans	98	92.18	294	MBD1539083.1
			Pseudarthrobacter polychromogenes	98	91.84	294	WP_188813142.1
			Pseudarthrobacter oxydans	100	91.28	294	WP_307464326.1
			Pseudarthrobacter defluvii	98	92.18	293	WP_307491979.1
Asphe3_40510	catechol 2,3-dioxygenase	pASPHE301	*Actinomycetota* bacterium	100	57.19	291	MDT7726568.1
			Rhodococcus wratislaviensis IFP 2016	100	56.46	293	ELB89778.1
			Carbobacillusaltaicus	100	45.64	298	PTQ55801.1
			Pseudaminobacter salicylatoxidans	100	38.61	301	PWJ76351.1
			Starkeya sp.	100	35.76	301	QCS37645.1
Asphe3_22000	4HB transporter	chromosome	*Subtercola lobariae*	90	50.93	470	GGF22968.1

Table S2. Oligonucleotides used for the RT-qPCR and PCR experiments.

Gene Locus Tag/ Gene Name	Oligonucleotide Designation	Oligonucleotide Sequence (5′→3′)	Product Size (bp)
**Asphe3_38860/*pca*34**	pca34dioxfor	CGTACCCATGGAAGAACCAC	248
	pca34dioxrev	GGTCAGGATGATGTCCCAGT	
**Asphe3_42380/*pca*45**	pca45dioxfor	ACACCTCGGCACTATTCACC	162
	pca45dioxrev	CGTTGTACACCAGGATGACG	
**Asphe3_35170/*cdo*12**	cat12dioxfor	AAACGGATACCCGGAAAGAG	198
	cat12dioxrev	GGGCGTTGTACTCCTCGTAG	
**Asphe3_40510/*cdo*23**	cat23dioxfor	AGCCAGTTCCACCACGATAT	182
	cat23dioxrev	CAATACTGGTTTCCGCCGAC	
**Asphe3_00060/*gyr*β**	gyrβfor	GGCTAACGACAATACAGATA	210
	gyrβrev	ACCACTTCATAAACAAGGT	
